# Editorial: Molecular interactions between crops and phytopathogens, volume II: Rice

**DOI:** 10.3389/fpls.2022.984072

**Published:** 2022-07-29

**Authors:** Guotian Li, Xiaodong Wang, Xiaojie Wang, Meixiang Zhang, Xiao-Ren Chen, Jianhui Wu, Jin-Ying Gou, Lisong Ma

**Affiliations:** ^1^State Key Laboratory of Agricultural Microbiology, Provincial Key Laboratory of Plant Pathology of Hubei Province, College of Plant Science and Technology, Huazhong Agricultural University, Wuhan, China; ^2^State Key Laboratory of North China Crop Improvement and Regulation, College of Plant Protection, Hebei Agricultural University, Baoding, China; ^3^State Key Laboratory of Crop Stress Biology for Arid Areas, College of Plant Protection, Northwest Agriculture and Forestry University, Xianyang, China; ^4^National Engineering Laboratory for Endangered Medicinal Resource Development in Northwest China, Key Laboratory of Medicinal Resources and Natural Pharmaceutical Chemistry of Ministry of Education, College of Life Sciences, Shaanxi Normal University, Xi'an, China; ^5^College of Horticulture and Plant Protection, Yangzhou University, Yangzhou, China; ^6^College of Agronomy, Northwest Agriculture and Forestry University, Xianyang, China; ^7^State Key Laboratory of Genetic Engineering, Ministry of Education Key Laboratory for Biodiversity Science and Ecological Engineering, Ministry of Education Engineering Research Center of Gene Technology, Institute of Plant Biology, School of Life Sciences, Fudan University, Shanghai, China; ^8^College of Horticulture, Hebei Agricultural University, Baoding, China

**Keywords:** *Oryza sativa*, fungal pathogen, multiple omics, hypersensitive response, plant hormone

Crop diseases reduce the yield and quality of agricultural products, seriously threatening human, animal and ecological heath (Dean et al., 2012). Rice (*Oryza sativa*), as the staple that feeds more than half of the world's population, is often afflicted with multiple devastating diseases, including rice blast caused by *Magnaporthe oryzae*, false smut caused by *Ustilaginoidea virens*, and sheath blight caused by *Rhizoctonia solani* (Ms, 2014). Besides these three aforementioned fungal diseases, there are severe bacterial diseases, including rice bacterial blight caused by *Xanthomonas oryzae* pv. *oryzae* (*Xoo*) and bacterial leaf streak caused by *Xanthomonas oryzae* pv. *oryzicola* (*Xoc*). Among these diseases, rice blast alone results in annual grain losses that are sufficient to feed more than 60 million people globally (Cook et al., 2011). Furthermore, rice false smut not only causes yield losses but also contaminates grains with mycotoxins (Sun et al., 2020), similar to notorious Fusarium head blight of wheat. To understand these diseases for better control strategies, researchers utilize bioinformatics, physiological, molecular, and biochemical tools to elucidate the complex pathogen-host interactions. In total, this Research Topic collects seven articles from researchers in the field and covers a broad range of subjects in the following three themes.

## Transcriptional analysis of the *M. oryzae*-rice pathosysterm

The early availability of genome sequences of *M. oryzae* and rice, including the recently published high-quality genomes (Qin et al., 2021; Wang et al., 2022; Yang et al., 2022), facilitates basic research of the *M. oryzae*-rice pathosystem. Equally important is the accurate genome annotation. In this special issue, Li Z. et al. used RNA-seq data to annotate the *M. oryza*e genome, leading to the identification of 3,374 additional genes, most of which encode long non-coding RNAs and often show alternative splicing events that could be important for growth, conidiation, and pathogenesis. RNA-seq has long been used in studying pathogen-host interactions and uncovering immunity-related genes. From transcriptomes of the rice cultivar Nipponbare infected by three different *M. oryzae* strains, Liang et al. identified thousands of conserved differentially expressed genes of rice and uncovered that overexpression of one such gene enhances rice immunity against blast. The above two papers provide rich genomic and transcriptomic information for the *M. oryzae*-rice interaction study.

## Molecular plant pathology of *M. oryzae* and *U. virens*

Three papers in this special issue cover molecular plant pathology of rice fungal pathogens *M. oryzae* and *U. virens*. Zhang et al. studied the *MoPCS60* gene that encodes a peroxisomal-CoA synthase in *M. oryzae*. The *Mopcs60* mutant is reduced in vegetative growth when the carbon source is limited to oleate and olive oil, and attenuated in virulence. The defects of the *Mopcs60* mutant are likely resulted from its defective fatty acid metabolism. The importance of lipids in *M. oryzae* pathogenesis is also evidenced by another recent publication, which shows that the phosphatidate phosphatase MoPah1 is important for fungal development and pathogenesis (Zhao et al., 2022). In another paper collected this special issue, Liu et al. showed that the calcineurin regulator MoRCN1 is required for full fungal virulence by *M. oryzae* through regulating the calcineurin pathway that often plays a key role in fungal development and pathogenesis. The *Morcn1* mutant is defective in appressorium formation, invasive growth, virulence, and suppressing reactive oxygen species (ROS) of the host. Mechanistically, MoRCN1 interacts directly with the calcineurin subunit A (MoCNA) in the calcineurin pathway of *M. oryzae*. Rice false smut recently emerges as a serious threat to rice production worldwide, particularly to those high-yielding cultivars. Effective resistance genes against rice false smut have not been widely deployed. Recently, molecular studies of *U. virens* pathogenesis provide valuable information for the development of novel control strategies. Recognition of fungal chitin by plant receptors triggers immune responses, which has been shown in multiple pathosystems (Gong et al., 2020). As a counteract, fungal pathogens have evolved various approaches to avoid the recognition. Li et al. elegantly showed that the chitin-binding protein UvCBP1 secreted by *U. virens* is important to attenuate chitin-triggered rice immunity including ROS burst, callose deposition, and expression of defense marker genes such as *OsPR10b*. When overexpressed, *UvCBP1* promotes fungal infection. Mechanistically, UvCBP1 competes with OsCEBiP-the rice chitin receptor-for binding to free chitin and suppresses rice immunity. These three papers together deepen our understanding of fungal-rice interactions.

## Plant immune inducers and phytohormones

Chemicals are an important means in disease control, but excessive uses of traditional fungicides lead to environmental pollution and health concerns. Wang et al. identified guanine-one nucleobase of the nucleic acids DNA and RNA-as a plant immune inducer from the crude extract of the endophytic fungus *Paecilomyces variotii*. Guanine induces a series of defense responses, including ROS burst, callose deposition, and activation of mitogen-activated protein kinases in Arabidopsis and rice, which enhance plant resistance to bacterial and fungal pathogens through a mechanism involved in ethylene and jasmonic acid signaling pathways. In another study, Xie et al. showed that salicylic acid (SA), jasmonate, and ethylene are important for rice resistance to the white tip nematode *Aphelenchoides besseyi*. Authors demonstrated that the SA-related genes are positively associated with rice resistance to the nematode by comparing expression of hormone-responsive genes in resistant and susceptible rice cultivars through quantitative real-time (qRT)-PCR assays. The exogenous application of analogs of the three plant hormones induces rice resistance to the nematode. In contrast, the hormone inhibitors make rice more susceptible to the nematode. The exogenous application results were further confirmed using genetic analyses. Taken together, results from these two papers demonstrate that plant hormones are important for rice resistance to bacteria, fungi and nematodes.

The results reported in this Research Topic provide key insights into pathogen-rice interactions. Yet, some cutting-edge techniques are awaiting researchers in this field to explore, including telomere-to-telomere (T2T) genome sequencing (Nurk et al., 2022), artificial intelligence, and synthetic biology, which will facilitate our deep understanding of pathogen-rice interactions and hence development of novel disease control strategies.

## Author contributions

All authors have participated in the article writing and have acted as coeditors of this Research Topic. All authors contributed to the article and approved the submitted version.

## Funding

GL was supported by the National Natural Science Foundation of China (32172373). XiaodW was supported by the Provincial Natural Science Foundation of Hebei (C2022204010, C2021204008) and State Key Laboratory of North China Crop Improvement and Regulation (NCCIR2021ZZ-4). XiaojW was supported by the Shaanxi Innovation Team Project (2018TD-004). MZ was supported by the National Natural Science Foundation of China (32072399, 31672008) and the Fundamental Research Funds for the Central Universities (GK202201017). X-RC was supported by the National Natural Science Foundation of China (31871907, 31671971) and Jiangsu Agriculture Science and Technology Innovation Fund (JASTIF) [CX(20)3125]. JW was supported by the Key R&D Program of Shaanxi Province in China (2021ZDLNY0-01). J-YG was supported by the National Natural Science Foundation of China (31972350). LM was supported by the Hundred Talents Program for the introduction of high-level overseas talents in Hebei Province (E2020100004).

## Conflict of interest

The authors declare that the research was conducted in the absence of any commercial or financial relationships that could be construed as a potential conflict of interest.

## Publisher's note

All claims expressed in this article are solely those of the authors and do not necessarily represent those of their affiliated organizations, or those of the publisher, the editors and the reviewers. Any product that may be evaluated in this article, or claim that may be made by its manufacturer, is not guaranteed or endorsed by the publisher.
